# Catecholaminergic Polymorphic Ventricular Tachycardia: Clinical Characteristics, Diagnostic Evaluation and Therapeutic Strategies

**DOI:** 10.3390/jcm13061781

**Published:** 2024-03-20

**Authors:** Abhinav Aggarwal, Anton Stolear, Md Mashiul Alam, Swarnima Vardhan, Maxim Dulgher, Sun-Joo Jang, Stuart W. Zarich

**Affiliations:** 1 Department of Internal Medicine, Yale New Haven Health, Bridgeport Hospital, Bridgeport, CT 06605, USA; abhinav.aggarwal@yale.edu (A.A.); mdmashiul.alam@bpthosp.org (M.M.A.); swarnima.vardhan@bpthosp.org (S.V.); 2 Division of Cardiology, Department of Internal Medicine, Yale New Haven Health, Bridgeport Hospital, Bridgeport, CT 06605, USA; anton.stolear@yale.edu (A.S.); dr.stuart.zarich@bpthosp.org (S.W.Z.); 3 Department of Internal Medicine, Nuvance Health, Norwalk Hospital, Norwalk, CT 06856, USA; maxim.dulgher@nuvancehealth.org; 4 Section of Cardiovascular Medicine, Department of Internal Medicine, Yale University School of Medicine, New Haven, CT 06519, USA

**Keywords:** catecholaminergic polymorphic ventricular tachycardia, arrhythmias, sudden cardiac death, beta-blockers, flecainide, left cardiac sympathetic denervation, gene therapy, electrophysiology, precision medicine

## Abstract

Catecholaminergic polymorphic ventricular tachycardia (CPVT) is a severe hereditary arrhythmia syndrome predominantly affecting children and young adults. It manifests through bidirectional or polymorphic ventricular arrhythmia, often culminating in syncope triggered by physical exertion or emotional stress which can lead to sudden cardiac death. Most cases stem from mutations in the gene responsible for encoding the cardiac ryanodine receptor (*RyR2*), or in the Calsequestrin 2 gene (*CASQ2*), disrupting the handling of calcium ions within the cardiac myocyte sarcoplasmic reticulum. Diagnosing CPVT typically involves unmasking the arrhythmia through exercise stress testing. This diagnosis emerges in the absence of structural heart disease by cardiac imaging and with a normal baseline electrocardiogram. Traditional first-line treatment primarily involves β-blocker therapy, significantly reducing CPVT-associated mortality. Adjunctive therapies such as moderate exercise training, flecainide, left cardiac sympathetic denervation and implantable cardioverter-defibrillators have been utilized with reasonable success. However, the spectrum of options for managing CPVT has expanded over time, demonstrating decreased rates of arrhythmic events. Furthermore, ongoing research into potential new therapies including gene therapies has the potential to further enhance treatment paradigms. This review aims to succinctly encapsulate the contemporary understanding of the clinical characteristics, diagnostic approach, established therapeutic interventions and the promising future directions in managing CPVT.

## 1. Introduction

Catecholaminergic polymorphic ventricular tachycardia (CPVT) is a rare genetic cardiac channelopathy characterized by unexplained syncopal episodes and sudden cardiac death (SCD) in patients with a structurally normal heart. These symptoms occur in the context of adrenergically mediated polymorphic ventricular arrhythmias, which are often triggered by emotional or physical stress. CPVT is linked to mutations in genes responsible for regulating calcium levels in cardiomyocytes. The inconspicuous presentation of CPVT poses challenges in diagnosis, therefore maintaining a high level of suspicion and familiarity with this condition is crucial. This review provides a comprehensive overview of the current state of knowledge on characteristic clinical findings, diagnostic approach and an adept understanding of evolving management strategies, given the high risk of recurrence of fatal arrhythmias in these patients.

## 2. History

In 1960, Norwegian cardiologist Dr. Knut Berg documented a case involving three sisters who experienced syncope during exercise or emotional stress, marking the initial recognition of CPVT [1]. The association between this condition and bidirectional ventricular tachycardia (VT) was first noted in 1975 [2]. The term “catecholaminergic polymorphic ventricular tachycardia” was introduced in 1978 [3] and in 1999, the first genetic mutation associated with CPVT was identified on chromosome 1q42-q43 [4]. Subsequently, this mutation was further characterized as a variant in the cardiac ryanodine receptor (*RyR2*) gene in 2001 [5]. 

## 3. Epidemiology

It is estimated that CPVT affects approximately 1 in 10,000 individuals [6,7]. CPVT is one of the most prevalent genetically identifiable causes of sudden unexplained death in individuals aged 1–35 years, especially in cases where autopsy results do not reveal structural heart defects. This is particularly significant if the death occurred during exertional activities such as swimming [8]. CPVT is considered responsible for up to 15% of unexplained SCD in young people [9].

The mean age of symptom onset ranges from 7 to 12 years [10], with over 60% of affected individuals experiencing their first syncopal episode or cardiac arrest by the age of 20 [2]. However, there are recognized atypical cases where symptoms emerge in the third or even fourth decade of life [6,11]. Approximately 30% of individuals diagnosed with CPVT have a family history of stress-related syncope, seizure or sudden cardiac death in relatives who were younger than 40 years old, with the prevalence of such events noted to be as high as 60% among families hosting *RyR2* mutations [10,12]. 

While CPVT affects both males and females equally, males usually present at a younger age (during childhood or adolescence) whereas females are more likely to present at an older age, with an average age of onset around 20 years [11].

## 4. Clinical Presentation

The most common initial presentation of CPVT is a syncopal episode occurring during exercise or acute emotional stress [2]. In some cases, underlying arrhythmias (predominantly VT) self-terminate, leading to spontaneous recovery. However, approximately one-third of VT cases may degenerate into ventricular fibrillation, resulting in SCD as the first symptom of the disease [13]. Minor symptoms might include palpitations and dizziness.

The physical examination of patients with CPVT frequently does not reveal any remarkable findings. Syncopal episodes at the initial presentation are often mistaken for vasovagal events or attributed to neurological triggers. A significant proportion of individuals may receive an incorrect diagnosis of epilepsy due to the similarity of their symptoms, which include seizure-like activity during syncope [14]. Given its significant contribution to SCD in young individuals, the identification and effective management of CPVT assumes paramount importance.

## 5. Genetics and Pathophysiology 

Disruptions in critical elements of intracellular calcium-induced calcium release from the sarcoplasmic reticulum during excitation–contraction coupling currently underlie the pathogenesis of CPVT. Numerous pathogenic gene variants have been identified in CPVT patients, with the *RyR2*-encoded cardiac ryanodine receptor/calcium release channel being the most commonly implicated, representing the predominant genetic subtype recognized as CPVT1 [5]. Typically, CPVT1 is inherited in an autosomal dominant fashion, however, rare instances of *RyR2*-related CPVT manifest with autosomal recessive transmission [15].

Most *RyR2* mutations associated with CPVT are missense variants (96%), leading to a gain-of-function of the *RyR2* channel resulting in abnormal calcium handling, characterized by excessive accumulation of diastolic calcium in the sarcoplasmic reticulum and its spontaneous release into the cytoplasm [16]. This leads to delayed afterdepolarizations and triggered activity, ultimately resulting in malignant ventricular arrhythmias, especially under conditions of physical stress or emotional excitement since catecholamine-mediated beta-adrenergic receptor activation can trigger premature sarcoplasmic reticulum calcium release [17,18]. This explains the frequent occurrence of CPVT episodes triggered by exercise or stress, as well as the efficacy of treatments focusing on the sympathetic nervous system, such as β-adrenergic receptor blockers and sympathetic denervation, in the management of the condition [19,20]. At a molecular level, the disease mechanisms include a range of abnormal cellular processes, from defective interactions between *RyR2* and its stabilizing proteins due to phosphorylation to impaired binding of calmodulin to *RyR2* [21,22,23,24]. 

CPVT-related *RyR2* variants are concentrated in four distinct mutational hotspot regions, which include exons groups 3–15, 44–50, 83–90 and 93–105. Only 10% of CPVT-related variants are found outside these hotspot regions [25]. The majority of monogenetic *RyR2*-related CPVT cases are attributed to de novo variants. Limited data indicate that individuals with de novo variants of *RyR2* mutation tend to experience symptoms of CPVT earlier and exhibit more severe phenotype traits compared to individuals with familial forms of CPVT [26].

However, mutations in *RyR2* gene have been identified in only up to 60% of patients. This suggests that other genes are also contributing to the development of this condition [27]. In a more recent analysis of published studies, additional pathogenic variants associated with CPVT have been identified. These variants include *CASQ2*, *TRDN*, *TECRL*, *KCNJ2*, *CALM1*, *CALM2* and *CALM3*. Although they are strongly linked to the condition, mutations in these genes are much less prevalent compared to the *RyR2* gene [28]. While *CASQ2* and *TRDN* have been linked to autosomal recessive forms of CPVT, *CALM1* and *CALM3*, akin to *RyR2*, are associated with an autosomal dominant form as outlined in Table 1. Unlike the gain of function pathogenic variants in *RyR2*, no genotype–phenotype correlations for these less common pathogenic variants have been identified. 

Homozygous or compound heterozygous mutations in *CASQ2* are observed in CPVT2, a subtype that represents a smaller proportion, accounting for less than 5% of CPVT index cases. *CASQ2* encodes calsequestrin-2 (*CASQ2*), a calcium-binding protein characterized by high-capacity, low-affinity properties, that is crucial for buffering calcium (Ca^2+^) within the sarcoplasmic reticulum. Mutations primarily result in reduced expression or complete absence of *CASQ2* in cardiac tissues, leading to compromised Ca^2+^ buffering, dysregulated *RyR2* activity and structural remodeling of the sarcoplasmic reticulum ultrastructure along with its constituent proteins [29]. Novel variants potentially associated with additional neurological manifestations have also been recently identified in patients with CPVT2 [30].

The estimated penetrance of CPVT may range between 63% to 78% [12,31,32]. *RyR2*-related disease exhibits a high penetrance rate (75–80%), indicating that asymptomatic individuals with *RyR2*-related CPVT constitute a minority [12]. Biallelic *CASQ2* pathogenic variants exhibit complete 100% penetrance in reported cases till date. However, the number of reported individuals with heterozygous *CASQ2*-, *KCNJ2*-, *CALM1*-, *CALM2*- or *CALM3*-related CPVT is quite limited which precludes a robust estimation of penetrance at this time [10].

The widespread availability of molecular genetic testing, incorporating gene-targeted and comprehensive genomic approaches, has led to an increasing identification of genes associated with sudden cardiac death (SCD) [33]. However, despite these advancements, most variants remain of uncertain significance (VUS) as per current American College of Medical Genetics and Genomics (ACMG) and Association for Molecular Pathology (AMP) recommendations [34]. While copy-number variants (CNVs) contribute to a small percentage of inherited channelopathies, their true impact is still unclear due to limited research on their frequency in the general healthy population and the absence of comprehensive genotype–phenotype studies, complicating their genetic interpretation. Nevertheless, recent studies advocate for the inclusion of CNV analysis in routine genetic diagnosis to enhance diagnostic yield [25]. In the context of CPVT, screening for CNVs has predominantly targeted the *RyR2* gene, with reported deletion frequencies ranging from 1.9% to 8.3% [25,35,36,37]. Conversely, a recent European study screening a panel of multiple SCD-related genes in 19 CPVT patients detected no CNVs [38]. It is proposed that while conventional genetic analysis typically identifies the underlying cause in approximately 60–65% of patients, with *RyR2* being the primary gene of interest, an exhaustive genetic analysis, encompassing single nucleotide variants (SNVs), small insertions and deletions (indels), as well as copy number variations (CNVs), holds promise in elucidating the potential cause in up to 70% of cases [33].

## 6. Diagnosis

A comprehensive clinical history is crucial for diagnosing CPVT. It is imperative to consider this diagnosis in individuals with a structurally normal heart and a normal resting ECG who have experienced one or more of the following symptoms during physical activity or sudden emotional stress: palpitations and lightheadedness, fainting or seizure-like episodes, unforeseen and sudden death and a familial history of juvenile SCD.

**Figure 1:**
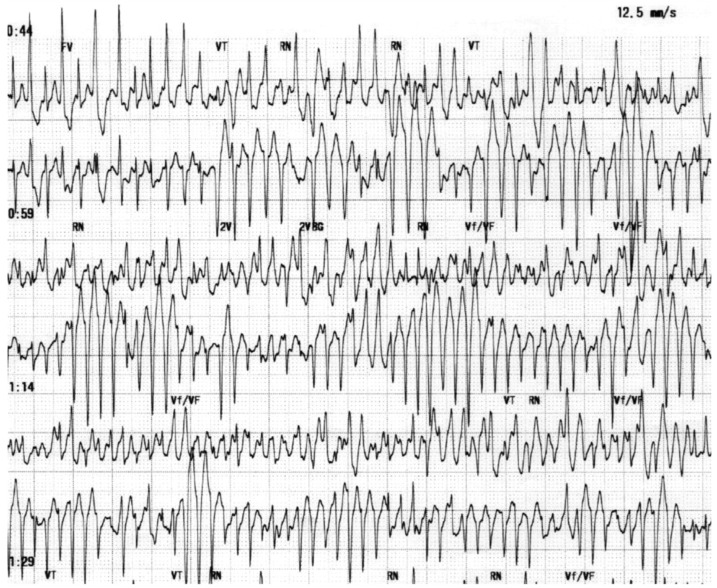
Polymorphic ventricular tachycardia (VT) during exercise test in adolescent female patient diagnosed with CPVT. Image reproduced from [39] under a creative commons [CC BY-NC-ND 4.0] license.

**Figure 2:**
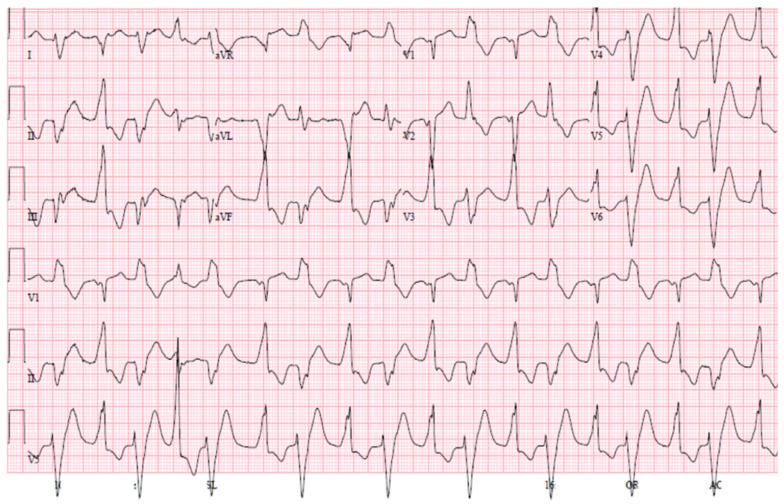
Twelve-lead ECG of a patient with CPVT characterized by bidirectional VT demonstrating QRS axis alternating with every other beat. Image reproduced from [40] under a creative commons [CC BY-NC-ND 4.0] license.

Individuals with CPVT may exhibit two distinct types of polymorphic VT. The first type (typical polymorphic VT) is characterized by constantly changing QRS morphology (Figure 1) [39]. The second type (bidirectional tachycardia) is marked by a 180° rotation of the QRS complex from one beat to the next (Figure 2) [40]. While the presence of bidirectional VT strongly suggests a possible diagnosis of CPVT, it is a rare occurrence. It is important to note that bidirectional VT may also manifest in other circumstances such as in cases of digitalis intoxication [41]. 

Typically, the premature ventricular complexes (PVCs) in CPVT are late-coupled and can exhibit left bundle branch block (LBBB) pattern with inferior axis or right bundle branch block (RBBB) pattern with superior axis. Certain features of PVCs identified during exercise testing can potentially assist in distinguishing CPVT from healthy controls, including a larger burden of PVCs, first appearance at higher workload, LBBB pattern and inferior axis, bigeminy or trigeminy at peak stress, QRS duration more than 120 milliseconds, coupling interval more than 400 milliseconds and disappearance in the first minute of the recovery [42]. LBBB pattern with inferior axis was noted to be the most sensitive and specific characteristic for detection of CPVT, suggesting right ventricular outflow tract (RVOT) as a common source of the initiating ectopy in pediatric patients with CPVT [43].

The exercise stress test is the primary diagnostic method used to diagnose CPVT. The stress test is conducted in a closely monitored environment and usually follows a standard Bruce protocol. During exercise testing, it is common to observe isolated and frequently monomorphic ventricular ectopy initially. With increasing exercise duration and workload, these isolated beats may transition to a bigeminal pattern. Subsequently, these may progress to polymorphic doublets or more complex ventricular arrhythmias [44]. The heart rate at which ventricular arrhythmias initiate is frequently consistent within an individual patient and generally occurs at a relatively predictable heart rate threshold. The test is stopped either when there is a growing frequency of PVCs with increasing exercise intensity or when the patient reaches their maximum exertion level. Both non-sustained and sustained VT can occur during either the stress or recovery phases.

Exercise provokes polymorphic or bidirectional VT in 63% of CPVT patients. Therefore, a negative provocative exercise stress test does not rule out the diagnosis [45]. In cases where a standard exercise study fails to demonstrate exercise-induced ventricular arrhythmias despite a high clinical suspicion for CPVT, a novel “burst” exercise testing protocol may be employed to uncover such arrhythmias. In a pilot study testing this protocol, the “burst” exercise stress testing was defined as abrupt high-intensity exercise at the immediate onset of testing, equivalent to the maximum stage achieved on previous standard exercise stress tests [46]. This rigorous exercise regimen was sustained until the point of volitional fatigue, the onset of cardiac symptoms or the emergence of more than three beats of non-sustained ventricular tachycardia (NSVT). The “burst protocol” was developed on the hypothesis that short, intense bursts of exercise may trigger an abrupt heart rate increase, along with a sudden catecholaminergic surge and a notable reduction in vagal tone thereby predisposing to arrhythmogenesis via calcium dysregulation and unmasking ventricular ectopy not previously evident on standard exercise testing. Additionally, it was also hypothesized that sudden exercise induces anaerobic metabolism, potentially serving as an additional contributing factor to arrhythmogenesis. A recent retrospective cohort study examined the relationship between the ventilatory anaerobic threshold (VAT), the point at which the body transitions from aerobic to anaerobic metabolism and the ectopy burden in patients with CPVT undergoing cardiopulmonary exercise testing (CPET). The study found that VAT consistently occurred at lower heart rates preceding the onset of ventricular ectopy at higher heart rates, suggesting a plausible role for the shift to anaerobic metabolism in the pathogenesis of arrhythmias in CPVT [47].

The exercise stress test serves not only as a diagnostic tool but also aids in stratifying patients based on their risk of arrhythmic events. Additionally, it can be used to assess the effectiveness of β-blocker therapy in lowering heart rate to levels below those associated with prior arrhythmias [48] and serial exercise testing can be used to titrate the medical therapy [49]. However, the effectiveness of β-blocker therapy in suppressing exercise-induced ventricular arrhythmias does not always translate to long-term therapeutic success [50].

Epinephrine infusion is used as an alternative diagnostic tool for patients who are unable to undergo exercise testing. The initial infusion rate of epinephrine starts at 0.05 to 0.1 mcg/kg/min, with subsequent incremental increases of 0.05 mcg/kg/min reaching a maximum of 0.20 mcg/kg/min. The diagnosis of CPVT is confirmed when an infusion of epinephrine triggers either non-sustained or sustained polymorphic VT with more than 10 PVCs per minute or new T-wave alternans. When compared with exercise testing, the epinephrine test demonstrated low overall sensitivity (28%) and high specificity (98%) [51].

For individuals who are unable to undergo an exercise test such as very young patients or those whose symptoms are related to emotion rather than exercise, Holter monitoring can be employed as an alternative diagnostic method. However, it is important to note that Holter monitoring is generally less sensitive than exercise testing for detecting arrhythmias in patients with CPVT [45].

For patients exhibiting clinical symptoms or familial history indicative of CPVT, genetic screening panels can aid in confirming the diagnosis. Given the ability of clinical genetic testing to identify potentially at-risk individuals before the onset of potentially lethal arrhythmias, the role of genetic testing in CPVT is considered to be robust. Genetic testing is recommended for CPVT-susceptibility genes—*RyR2*, *CASQ2*, *CALM1-3*, *TRDN* and *TECRL*—in all probands with a definitive clinical diagnosis of CPVT and may be considered in individuals with idiopathic ventricular fibrillation (VF) upon identification of an adrenergic trigger. Current Heart Rhythm Society and European Heart Rhythm Association guidelines recommend diagnostic molecular genetic testing in all cases of clinically suspected CPVT followed by cascade screening of potentially at-risk first-degree relatives of a proband if either a pathogenic variant or likely pathogenic variant is identified [52,53]. In cases where the family-specific variant(s) are not known, it is recommended that all first-degree relatives of an affected individual undergo evaluation, which includes resting electrocardiogram (EKG), Holter monitoring, echocardiography and exercise stress testing [10].

Table 2 provides a concise overview of the diagnostic criteria for CPVT as outlined in the Heart Rhythm Society consensus statement [54]. 

Giudicessi et al. have recently proposed a diagnostic scorecard aimed at objectively measuring the pre-test probability of CPVT as outlined in Table 3 [55]. Given the inherent challenges associated with the malignant nature and often elusive diagnosis of CPVT, this tool offers an efficient and semi-quantitative assessment of clinical parameters. It aids in the integration of robust disease-specific metrics into the adjudication process of *RyR2* variants with uncertain significance (VUS), potentially reducing the VUS rate significantly from 48% to 7%, thereby improving the ability of current variant classification and reporting standards to distinguish between pathogenic *RyR2* variants misclassified as VUS and true VUS or benign *RyR2* rare variants. Utilizing this approach, patients with a CPVT diagnostic score of 3.5 (without genetic test results) exhibit a likelihood of CPVT1 (i.e., RyR2-mediated CPVT) of at least 60% [55].

## 7. Prognosis and Risk Stratification

The average time from the initial symptom report to diagnosis was 2 years (±0.8 years) [11]. Without being recognized and properly treated in a timely manner, CPVT can be highly lethal. Approximately 30% of affected individuals have experienced at least one cardiac arrest, while up to 80% have had one or more syncopal episodes prior to diagnosis [10]. The potential lethality of CPVT is underscored by mortality rates ranging from 30% to 50% by the age of 35. Furthermore, approximately 12% of autopsy negative sudden unexplained deaths in young individuals, as well as few cases of sudden infant death syndrome (SIDS), have been linked to CPVT [56].

Risk stratification for cardiac events in CPVT patients remains ambiguous, particularly in asymptomatic individuals or mutation carriers, with a lack of established markers to discern high- or low-risk patients, necessitating reliance on clinical phenotype for risk assessment and mirroring approaches in other inherited channelopathies. In patients with CPVT, independent predictors for adverse cardiac events include a younger age at the time of diagnosis, proband status, multiple genetic variants, autosomal recessive inheritance, history of prior aborted cardiac arrest and the absence of β-blocker therapy or incomplete symptom suppression despite pharmacological treatment [11,19,57,58,59]. No discernible disparities based on gender or age have been identified concerning the risk of arrhythmias in the pediatric populations as of now [60]. 

In a retrospective analysis, the presence of early repolarization pattern (ERP) was associated with an increased likelihood of symptomatic presentation [61]. While the detection of NSVT during exercise testing has been linked to poorer outcomes, instances of cardiac arrest have also been documented in CPVT patients even in the absence of arrhythmias during stress tests [62]. Furthermore, while the presence of couplets or more consecutive PVCs has shown predictive value for future cardiac events, invasive electrophysiological testing with programmed electrical stimulation has not proven to be valuable for risk stratification in these patients due to the rarity of inducible arrhythmias [19].

No genetic modifiers influencing the risk of cardiac events in CPVT have been identified [12,63]. Additionally, efforts to establish genotype–phenotype correlations for risk stratification, regardless of the presence or absence of a mutation or polymorphism, have not yielded clinically useful insights for identifying patients at risk of aborted cardiac arrest (ACA)/sudden cardiac arrest (SCA) [19,64]. Nevertheless, [12] found a higher burden of NSVT among patients harboring mutations in the C-terminal channel-forming domain of *RyR2* compared to those with mutations in the N-terminal or the central domain.

## 8. Therapeutic Strategies

Effective management of individuals with CPVT necessitates a comprehensive and tailored approach aimed at mitigating the primary manifestations of the condition. Precision in decision-making becomes paramount, delicately balancing the prevention of ventricular arrhythmias with the optimization of overall quality of life. Holistic management strategies for a typical CPVT patient involve a synergistic application of diverse interventions aimed at reducing the occurrence of life-threatening arrhythmic events. These interventions primarily include lifestyle modifications, pharmacological treatments, left cardiac sympathetic denervation and implantable cardiac defibrillators (ICD) [65,66]. 

**Figure 3:**
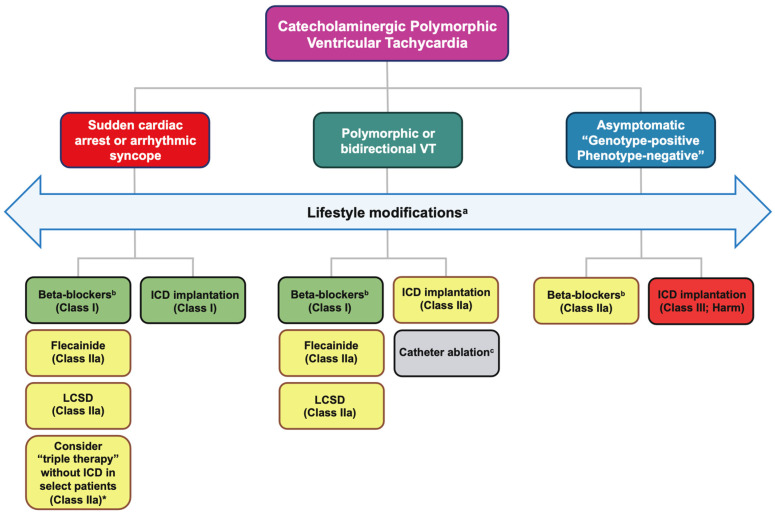
Schematic flowchart of current treatment strategies in CPVT #. ^a^ Lifestyle modifications: avoidance of competitive sports, avoidance of strenuous exercise, routine exercise stress tests to ensure adequate arrhythmia control, avoidance of stressful/emotional environments, dehydration, electrolyte disturbances or hypothermia. ^b^ Non-selective β-blockers: nadolol preferred (propranolol preferred where nadolol unavailable). ^c^ Currently not advocated by guidelines but represents a promising therapeutic strategy. CPVT: catecholaminergic polymorphic ventricular tachycardia; ICD: implantable cardioverter defibrillator; LCSD: left cardiac sympathetic denervation; VT: ventricular tachycardia. *#* Adapted from [67] and * Adapted from [68].

For patients exhibiting symptoms and a positive phenotype, a consensus exists on the imperative need for aggressive pharmacological management. In instances where optimal medical therapy fails to alleviate persistent symptoms or exercise-induced ventricular arrhythmias, non-pharmacological interventions should be contemplated selectively. Device therapy with ICD is recommended for secondary prevention in individuals requiring additional measures beyond pharmacological and non-pharmacological interventions. Figure 3 describes the schematic flowchart of current treatment strategies in CPVT. 

### 8.1. Lifestyle Modifications

Given that CPVT represents a cardiac channelopathy particularly susceptible to exercise-induced triggers, with catecholamine release being a primary proarrhythmic factor, lifestyle modifications and supportive care assume utmost significance for all affected individuals. The European Society of Cardiology (ESC) guidelines advocate a universal directive to restrict or abstain from strenuous physical activity, competitive sports and stress-inducing environments for both symptomatic probands and asymptomatic genotype-positive relatives [67]. Recommendations for competitive sports participation in asymptomatic athletes with concealed channelopathy are outlined, although these guidelines are based on expert opinion and lack comprehensive empirical validation [69]. 

The optimal restriction of exercise remains unspecified, but many centers advocate limiting the maximum allowable heart rate to the point where ventricular arrhythmias manifest during exercise stress testing. Nevertheless, the necessity for stringent limitations has been questioned [70,71]. A small observational study revealed comparable rates of arrhythmic events between well-treated athletes and non-athletes [71]. While data on risk stratification for identifying high-risk subsets is insufficient, individuals with concomitant neurodevelopmental disorders appear to constitute a high-risk subgroup with a malignant phenotype [72]. Given that a majority of patients are children and adolescents, the prolonged restrictions over physical activity need to be carefully weighed against prospective ramifications including but not limited to obesity, growth retardation, psychological challenges and impaired interpersonal relationships [73,74]. 

The optimal long-term approach involves collaborative decision-making to formulate personalized recommendations aimed at diminishing adrenergic stimulation [73]. According to the recent position statement from the European Association of Preventive Cardiology (EAPC) and the European Heart Rhythm Association (EHRA), the following guidelines are proposed for individuals with CPVT [73]:

1. Competitive and intensive leisure-time sports are strongly discouraged.
2. Consideration of low-intensity to moderate leisure-time sports is permissible if the patient remains asymptomatic for a minimum of three months and stress tests reveal the absence of any ventricular ectopy/arrhythmia even in those with an ICD.
3. Gene carriers with a pathogenic CPVT mutation lacking overt symptoms should be managed akin to patients with manifest CPVT, permitting only low-intensity sports.
4. Follow-up protocols should incorporate stress tests and/or continuous electrocardiogram (ECG) monitoring (Holter) during low-intensity leisure-time sports activities to ensure control of exercise-induced ventricular arrhythmias.
5. Recommendations include avoidance of stressful/emotional situations, dehydration, electrolyte disturbances or hyperthermia.

### 8.2. Medical Management 

#### 8.2.1. Beta-Blockers

Beta-blockers represent the cornerstone in the therapeutic approach to prevent arrhythmic events in individuals with CPVT [65]. It is strongly recommended to initiate treatment with β-blockers as the first-line therapy for symptomatic patients given the intimate connection between β-adrenergic stimulation and the pathophysiology of CPVT. The lifelong administration of β-blockers without intrinsic sympathomimetic activity forms the unequivocal foundation of therapeutic management [60,67,75].

In light of the elevated risk of potentially fatal cardiac events, guidelines advocate a Class Ic recommendation for symptomatic patients [65]. The titration of β-blockers to the maximum tolerated dose is advocated for all patients with a clinical diagnosis. Furthermore, β-blockers are recommended for use in asymptomatic concealed carriers of pathogenic mutations (genotype-positive phenotype-negative patients) with a Class IIa recommendation, considering documented cases of cardiac arrest within this population [62,65].

Non-selective β-blockers are the preferred pharmacological agents for managing symptomatic patients with CPVT. Extensive evidence suggests that nadolol, administered at a daily dosage of 1 to 2 mg/kg, stands out as the preferred β-blocker, demonstrating superiority over its β1-selective counterparts [76]. The precise mechanism accounting for this disparity remains incompletely elucidated. Recent investigation underscores the significantly heightened proclivity for arrhythmic events associated with β1-selective β-blockers, particularly in symptomatic children with CPVT, when compared to nonselective β-blockers, specifically nadolol (hazard ratio [HR] 2.04, *p* = 0.002) [77]. Similarly, in a study of 216 RyR2 variant-carrying CPVT patients, the risk of arrhythmic events was notably elevated in those prescribed β1-selective β-blockers compared to nadolol (HR 5.8, *p* = 0.001), with no significant differences observed between nadolol and propranolol [78]. In regions where nadolol is unavailable, propranolol may serve as a viable alternative.

Emphasizing strict adherence to β-blocker therapy is vital as a significant proportion of fatal arrhythmic events result from medication non-compliance [79,80]. A recent study from a representative international cohort of CPVT patients found that 15% patients are non-adherent to their CPVT medication with concerns about medication, flecainide monotherapy and female sex being independently associated with non-adherence [81]. Another study revealed an increase in cardiac events during evening hours, with 60% of these events attributed to medication non-adherence [82]. This underscores the pivotal role of medication adherence in ameliorating adverse outcomes in these patients [57].

In contrast to the observed detrimental impact of the absence of β-blocker therapy on outcomes [19], a recent multinational cohort study within the international CPVT registry revealed that 10% of patients necessitate a β-blocker-free treatment strategy prompted by the occurrence of unacceptable side effects [83]. This may be particularly true in patients with accompanying sinus node dysfunction, limiting the use of therapeutic doses to prevent fatal arrhythmias [84,85]. 

The study concluded that, following meticulous risk assessment, viable and efficacious β-blocker-free treatment strategies can be accomplished without the need for an ICD, for asymptomatic patients exhibiting an absent or negligible stress test phenotype, characterized by the absence of bigeminy, couplets or more complex ventricular ectopy [83]. However, it is essential to note that these findings warrant further substantiation through larger-scale studies or trials before definitive conclusions can be drawn.

#### 8.2.2. Flecainide

Despite optimal treatment with β-blockers and medication adherence, up to 30% patients with CPVT may require additional therapy due to recurrent symptoms or significant arrhythmias during follow-up exercise tests [16,86]. The initiation of arrhythmias depends on both heart rate and sympathetic stimulation, suggesting that combination therapy is crucial for improved outcomes [87]. In cases of breakthrough events despite β-blocker therapy, additional intervention with medications like flecainide is warranted [49,88].

Flecainide, a class IC anti-arrhythmic agent, effectively reduces ventricular arrhythmias in symptomatic CPVT patients already on the maximum tolerated dose of β-blockers [88]. While the primary mechanism of action in CPVT has been a subject of debate, recent studies suggest that its efficacy may stem from *RyR2* blockade, potentially inhibiting ryanodine-receptor (*RyR2*)-mediated sarcoplasmic reticulum calcium release [89,90]. Another contributing mechanism involves Na^+^ channel blockade, reducing the frequency of triggered activity leading to an associated increase in the arrhythmogenic threshold [91,92].

Recent ESC guidelines endorse flecainide, prescribed at 2–3 mg/kg/day, as the primary additional therapeutic option in cases of incomplete arrhythmia control [67]. For patients experiencing breakthrough arrhythmic events on β-blockers or persistent ventricular arrhythmias during exercise testing, supplementation with flecainide is recommended as Class IIa recommendation [67]. The conventional daily dose of flecainide typically ranges between 100–300 mg.

In the solitary randomized placebo-controlled crossover study including only 14 patients, initially designed with VT or appropriate ICD therapy as the primary endpoint, a protocol modification ensued focusing solely on the secondary endpoint of ventricular arrhythmias during exercise testing due to enrolment challenges. The study revealed the superiority of adding flecainide to β-blocker therapy over maximally tolerated β-blocker therapy alone in reducing exercise-induced ventricular arrhythmias in CPVT patients. Notably, no participants experienced couplets or NSVT during exercise testing in the flecainide group [88]. In a recent multinational retrospective cohort study involving 247 patients diagnosed with a clinical and/or genetic diagnosis of CPVT, the adjunctive use of flecainide with β-blockers demonstrated a significant reduction in arrhythmic events, defined as SCD, SCA, appropriate ICD shocks and arrhythmic syncope. The observed effect was consistent across the entire cohort, among symptomatic patients, and notably in those experiencing breakthrough arrhythmic events while on β-blocker therapy [93].

While the utilization of flecainide as a monotherapy has been documented in select cases and a small observational study with moderately positive outcomes, its efficacy is generally deemed less robust compared to combination therapy with a β-blocker [94]. Overall, the evidence supporting flecainide remains limited, particularly due to the absence of randomized control trials. Furthermore, a comprehensive understanding of long-term outcomes, optimal dosing and the potential role of flecainide as a primary intervention for preventing life-threatening arrhythmic events is currently lacking. The existing assessment of efficacy primarily relies on evaluating the burden of ventricular arrhythmias during exercise tests. Therefore, robust, large-scale studies are imperative to substantiate the effectiveness of flecainide in the absence of β-blockers, particularly for its long-term efficacy in preventing cardiac events.

#### 8.2.3. Other Medications

Alternative antiarrhythmic agents such as propafenone, verapamil and ivabradine have been investigated. Propafenone, a class IC sodium channel blocking agent, has seen limited application in the context of CPVT and has not been a primary focus of investigative research. The existing evidence is scant and has been primarily derived from case reports [95,96]. Comprehensive studies are warranted to assess the efficacy of propafenone within this patient population, discerning its potential as an alternative in situations where flecainide is either unavailable or not well-tolerated.

Evidence supporting the utilization of non-dihydropyridine calcium channel blockers is relatively limited and verapamil is seldom employed for management of CPVT in clinical practice. While a few preliminary small-scale studies cautiously expressed optimism regarding the supplementary efficacy of verapamil alongside β-blockers, extended follow-up data failed to demonstrate any significant benefits [97,98,99].

Ivabradine, a hyperpolarization-activated cyclic nucleotide-gated (HCN) channel blocker, has been sporadically tested in combination with nadolol or flecainide and was well-tolerated [100]. However, its potential use may be hindered by the inherent sinus bradycardia observed in many patients. Animal models investigating ivabradine revealed no reduction in delayed afterdepolarizations in isolated cardiomyocytes or delayed ventricular arrhythmias in their mouse model. Consequently, the authors concluded that ivabradine should not be considered as a treatment for CPVT [101].

Dantrolene, an alternative ryanodine receptor blocker, conventionally utilized for skeletal muscle ryanodine receptors (*RyR1*) in the treatment of malignant hyperthermia, has demonstrated the ability in animal models to attenuate abnormal calcium handling by binding to *RyR2*-mutated cardiomyocytes [102,103,104]. However, no human studies have been conducted to explore its therapeutic efficacy and safety.

### 8.3. Non-Pharmacological Therapies 

#### 8.3.1. Left Cardiac Sympathetic Denervation

Left cardiac sympathetic denervation (LCSD) has emerged as an efficacious anti-fibrillatory intervention for CPVT patients [105,106]. The initial success of the procedure for CPVT was documented in 2008, where three patients, previously refractory to medications, achieved symptom-free status following the procedure [105]. This surgical procedure involves thoracoscopic dissection of the lower two-thirds of the left stellate ganglion along with several upper thoracic ganglia (typically T2 through T4) [107,108,109]. It serves as a highly effective therapeutic option, disrupting the primary source of sympathetic stimulation to the heart.

In contrast to numerous antiarrhythmic interventions, the precise mechanisms of action of LCSD have undergone meticulous scrutiny and comprehension. Left cardiac sympathetic denervation elevates the VF threshold, introducing a heightened resistance to cardiac fibrillation [110]. This effect is arguably its most pivotal, exerting a more pronounced influence on VF onset than on the brief occurrence of torsades-de-pointes (TdP) ventricular tachycardia, the signature arrhythmia in long QT syndrome (LQTS) [111]. Additional antiarrhythmic effects encompass the prolongation of ventricular refractoriness [112], thereby diminishing the propensity for re-entrant arrhythmias [113]. Furthermore, the reflexive augmentation of cardiac vagal efferent activity, with its well-known antiarrhythmic effect, contributes to the multifaceted impact of LCSD [114,115].

LCSD is suggested as an adjunctive therapy for patients for whom pharmacological interventions prove ineffective or impractical. Guidelines advocate considering LCSD in individuals diagnosed with CPVT when the combination of β-blockers and flecainide at therapeutic dosages lacks efficacy, is poorly tolerated or is contraindicated (Class IIa recommendation) [67]. However, LCSD is not curative and is not recommended as a standalone therapy for CPVT patients, as one-third of patients still experience recurrences of arrhythmia [20].

In the largest multicenter follow-up series involving 63 patients, a substantial decrease in the occurrence of major cardiac events was observed, declining from 86% (54 out of 63) to 21% (13 out of 63; *p* < 0.001) over a median follow-up period of 37 months. Within the subgroup of patients who remained symptomatic despite optimal medical therapy prior to LCSD, only one-third experienced a recurrent cardiac event following the procedure. The mean annual event rates exhibited a remarkable 92% reduction, decreasing from 3.4 (95% CI: 3.2–3.7) pre-LCSD to 0.5 (95% CI: 0.4–0.6) post-LCSD [20]. This study suggests that in cases of persistent syncope despite optimal medical therapy, LCSD may be a preferable alternative to an ICD and could potentially complement it in patients experiencing recurrent shocks. Expert opinions now advocate implementing “triple therapy” (i.e., nadolol, flecainide and LCSD) in patients who have experienced a sentinel SCA prior to diagnosis [107]. This is particularly relevant in this challenging population where arrhythmia breakthroughs can occur even with a single missed medication dose, and LCSD adds an additional layer of protection in such circumstances.

The addition of right cardiac sympathetic denervation (RCSD) to LCSD, forming a complete bilateral cardiac sympathetic denervation (CSD), is beneficial when LCSD alone is ineffective for arrhythmia control. Expert opinion suggests considering RCSD as an interim step before ICD implantation in situations where LCSD does not offer adequate protection, as well as when patients continue to experience appropriate ICD shocks post-LCSD [107]. Limited case reports have also demonstrated a positive impact of renal sympathetic denervation on ventricular arrhythmias associated with CPVT. However, additional research is essential to clarify the risks and benefits of this approach in CPVT [116,117].

Complications associated with LCSD such as ptosis, Horner syndrome, harlequin flushing post-aerobic exercise, emotional excitement, pneumothorax and neuropathic pain have been documented [107,118]. Despite initial concerns about potential impact on quality of life, such complications are infrequent and usually transient. Remarkably, LCSD has demonstrated an improvement in the quality of life, particularly among patients with LQTS and CPVT who already have an ICD [119].

#### 8.3.2. Implantable Cardioverter-Defibrillators (ICDs)

ICDs have traditionally served as the cornerstone of therapy for CPVT patients with a history indicative of a high risk of sudden cardiac death, particularly in cases where β-blocker therapy proves inadequate. Both the AHA/ACC/HRS guidelines and the recently updated ESC guidelines maintain a Class I indication for patients with a history of ACA [67,120]. The ESC Guidelines additionally provide a Class IIa recommendation for ICD placement in patients who experience arrhythmogenic syncope and/or demonstrate documented bidirectional/polymorphic ventricular tachycardia while on the highest tolerated β-blocker dose and on flecainide [67].

The research surrounding the decision-making for ICDs is contentious and is primarily derived from observational studies with a notable absence of data from randomized controlled trials (RCTs). Numerous drawbacks associated with ICD therapy have been reported, indicating that ICDs are not devoid of the risk of substantial morbidity in this specific population subset. One review indicated that 85% of CPVT patients encountered complications related to ICDs during a mean follow-up of 54 ± 43 months [121].

ICD shocks, whether appropriate or inappropriate, have the potential to provoke or exacerbate arrhythmias due to the catecholamine surge induced by the associated pain and fear. This poses a risk of a potentially lethal electrical storm driven by shock therapy that intensifies the adrenergic drive [122,123]. Inappropriate ICD shocks occur more frequently, reported in up to 20–30% of patients with CPVT, compared to other cardiac channelopathies [45,124]. Nearly half of the inappropriate shocks can be attributed to frequent atrial tachyarrhythmias and episodes of non-sustained ventricular tachycardia that spontaneous resolve before ICD discharge [121,125,126]. The efficacy of ICD shocks in CPVT appears to be contingent on the arrhythmia mechanism. Shocks delivered for polymorphic ventricular tachycardia and bidirectional ventricular tachycardia are generally unsuccessful, while the conversion success rate for ventricular fibrillation is extremely high [126]. A study indicated that 99% of shocks for VT failed despite being appropriate, whereas 94% of shocks for VF were successful [127]. Additionally, patients undergoing ICD implantation are typically young, increasing their vulnerability to complications throughout their lifetime given the prolonged exposure to potential risks associated with ICD therapy [127]. Compounding these concerns is the observed inadequacy in the administration of optimal medical treatment to a substantial proportion of ICD recipients.

In a recent meta-analysis examining 53 studies with 1429 CPVT patients (35% of whom underwent ICD implantation) the follow-up revealed that 40% of patients experienced at least one appropriate shock, 21% received at least one inappropriate shock, 20% encountered an electrical storm (defined as three or more sustained episodes of ventricular tachycardia, ventricular fibrillation or appropriate shocks from an ICD within 24 hours), and 60% (7) deaths were attributed to ICD-associated incessant VTs [127]. A recent study from the international CPVT registry investigated 136 patients with a sentinel sudden cardiac arrest and found that the ICD did not confer a survival benefit. Over a median follow-up of 4.8 years, the composite outcome of SCD, sudden cardiac arrest, syncope or appropriate ICD shocks occurred in 47% of patients with an ICD compared to only 15.8% of patients without an ICD. It is worth noting that the patients without ICD received a higher proportion of nadolol/propranolol than patients with an ICD, potentially confounding the results. A significant proportion of patients, both with and without an ICD, who experienced nonfatal arrhythmic events during follow-up did not receive optimal treatment. Additionally, inappropriate ICD shocks were high, observed in 24% cases, and other device-related complications were observed in 29% of patients [128]. Strict adherence to guideline-directed therapy without an ICD may provide adequate protection in these patients without all the potential disadvantages of an ICD. The study even recommended considering forgoing ICD placement, emphasizing strict adherence to guideline-directed therapy as a potentially effective and advantageous approach, that provides sufficient protection without the potential drawbacks associated with an ICD.

The data presented highlights the dual burden of ineffectiveness and pro-arrhythmia with ICD therapy, leading to substantial medical complications and psychological burden, particularly in pediatric populations. Consequently, the utility of ICD implantation for CPVT patients is now deemed controversial and of questionable value, especially in asymptomatic patients [129]. Therefore, adherence to optimal pharmacological therapy with β-blockers and flecainide, meticulous ICD programming with high cut-off rates for heart rate recognition and maximizing time to detect episodes of polymorphic VT with a high likelihood of self-termination, are crucial to prevent inappropriate shocks and potential proarrhythmic effects [125]. It is advisable to program a singular VF zone at a higher threshold (ranging from 230 to 300 beats per minute) for VF detection rather than VT, thus reducing the likelihood of shocks triggered by ventricular ectopy and transient non-sustained episodes of bidirectional VT and polymorphic VT. The efficacy of programmed antitachycardia pacing (ATP) in restoring sinus rhythm in CPVT patients remains inconclusive [125]. Furthermore, given the typical absence of bradycardia pacing requirements in CPVT, a single-chamber ICD is generally deemed adequate [130]. 

In recent years, subcutaneous implantable cardioverter-defibrillators (S-ICDs) have emerged as a promising alternative for managing inherited arrhythmias. However, existing studies have primarily focused on patients with long QT syndrome (LQTS) and Brugada syndrome, with limited investigation into their application in CPVT [131,132]. Initial concerns persist regarding the use of S-ICDs in inherited arrhythmia populations, particularly related to the challenges in programming variables such as time to therapy and time to redetection [125]. Additionally, it is recognized that inappropriate shocks due to cardiac oversensing are more prevalent with S-ICDs compared to transvenous ICDs (TV-ICDs) [133]. A recent pooled analysis comparing complication rates associated with S-ICD placement versus transvenous ICD in CPVT patients revealed that all complications were attributed to inappropriate shocks resulting from supraventricular arrhythmias, T/R-wave oversensing or electrode defects. The study concluded that although lead failure or fracture complications are rare, the incidence of complications linked to inappropriate shock events is notably high with S-ICDs [134].

Emerging evidence suggests improved efficacy and safety of S-ICDs with contemporary devices and programming in order to lower rates of inappropriate shocks compared to many transvenous devices. This improvement may be attributed to the integration of high-rate cut-offs and advanced electrogram filtering algorithms [135]. In a study using the EFFORTLESS-SICD registry, outcomes in 199 channelopathy patients (including 5.5% with CPVT) were compared to those with structural heart disease and with a previously reported meta-analysis of transvenous ICDs in channelopathies. The study found similar S-ICD efficacy and a reduced incidence of inappropriate shocks in channelopathy patients compared to those with structural heart disease. Similar rates of inappropriate shocks were achieved with S-ICD programming set to higher rates for channelopathy patients [136].

In summary, despite strong endorsement from guidelines, there has been a paradigm shift in the threshold for ICD implantation in CPVT, reflecting a change in attitude based on recent evidence. Expert opinions now advocate reserving ICD placement as a last resort, specifically for rare cases where the combination of nadolol/propranolol, flecainide and LCSD has proven inadequate [129]. An emerging perspective suggests that, given the growing evidence of the effectiveness of LCSD, ICD placement should not be undertaken without prior LCSD, and such therapy should be approached with judicious consideration [70]. In fact, the recent PACES guidelines offer a Class IIa recommendation, suggesting that pharmacologic therapy or cardiac sympathetic denervation without an ICD may be considered as an alternative when aborted sudden cardiac arrest is the initial presentation of CPVT [68]. Recent contemporary data indicates a growing potential for the utilization of S-ICDs in inherited arrhythmias. Nevertheless, comprehensive data evaluating the effectiveness and safety of S-ICDs in individuals with CPVT is imperative before issuing conclusive recommendations regarding their application in this specific patient subset to mitigate the risk of sudden cardiac death.

#### 8.3.3. Catheter Ablation

Catheter ablation of PVCs inducing VF holds promise as a therapy for preventing these fatal arrhythmias in CPVT patients. While prior efficacy data stemmed from sporadic cases, exploratory research into its therapeutic effectiveness has recently gained momentum [137,138,139]. A recent case series assessing long-term efficacy in 5 female patients, with an average follow-up of 71 months, revealed a reduction in the frequency of ventricular arrhythmic events post-ablation. However, 80% of ablated patients experienced recurrent ventricular arrhythmias, necessitating intervention with an ICD or an external defibrillator. The study reported an average duration of approximately four years from ablation to the recurrence of ventricular arrhythmic events [140].

The most extensive analysis to date (involving 14 Japanese patients) explored long-term electrophysiological features and ablation outcomes in CPVT patients [141]. The findings suggested that catheter ablation of polymorphic VT/VF-triggering PVCs is a safe and viable adjunctive treatment for those who cannot receive flecainide, potentially attenuating syncope burden and notably, ameliorates exercise-related ventricular arrhythmias. The study identified a close association between induced PVCs during catecholamine stress testing and the onset of VT/VF events. Contrary to the prior studies that reported the right ventricular outflow tract as the primary source of CPVT arrhythmias, this study observed that the predominant PVCs triggering polymorphic VT/VF originated primarily from the left ventricle (LV). Specifically, the LV basal anterior wall and LV septal area were noted to be the main sites of triggering beats, with approximately one-third of cases demonstrating biventricular triggering beats. Successful elimination of these triggering beats resulted in non-inducibility of VT/VF events in over 90% of patients, with nearly 60% remaining free from syncope during follow-up. Syncope events predominantly occurred beyond one-year post-ablation, and approximately 20% of patients experienced only one episode throughout the follow-up period. The study also found that the induction of non-triggering PVCs after ablation indicated a high risk of syncope recurrence.

This suggests that while catheter ablation may decrease the risk of VAs, it does not eliminate the arrhythmogenic substrates in CPVT patients entirely [141]. β-blockers continue to be the therapeutic cornerstone for all patients, regardless of successful trigger beat ablation. For patients exhibiting induction of non-triggering PVCs post-ablation, early interventions such as LCSD or implantation of an ICD may be warranted [141].

### 8.4. Potential Future CPVT Therapies

The advancements in precision medicine hold great promise in managing inherited channelopathies by enabling development of tailored therapies for specific gene defects [27]. This approach has the potential to significantly impact healthcare systems by alleviating the social and economic burden associated with inadequate prevention strategies. Recent studies highlight the emergence of gene therapies targeting underlying mechanisms to control hazardous arrhythmias and impede disease progression, something that is particularly evident in CPVT [142]. The recognition of distinct forms of CPVT with diverse etiologies such as dominant mutations in *RyR2* or recessive mutations in *CASQ2* mark a pivotal advancement in embracing rapidly evolving precision medicine strategies, showcasing promising efficacy in experimental models [27].

Gene therapy, a broadly defined therapeutic approach involving the introduction of genetic material into cells, encompasses various modalities such as viral vectors, oligonucleotides and modified mRNA [143]. Multiple gene therapy strategies are currently under exploration for CPVT, each distinguished by distinct potential activities, specificity, pharmacokinetic properties and translational challenges, which aim to counteract the molecular repercussions associated with a spectrum of causative variants.

The utilization of recombinant viral vectors, a prominent gene transfer method, seeks to enhance the expression of a specific gene, the diminished expression of which is linked to the pathology of the disease. An illustrative instance involves the *CASQ2*-dependent form of CPVT, where the delivery of a functional wild-type (WT) *CASQ2* seeks to replace the absent or dysfunctional gene, hypothesized to reinstate the normal cascade of Ca^2+^-induced Ca^2+^ release [144,145,146]. Allele-specific silencing utilizes RNA interference to diminish the expression of a mutant allele by exerting a dominant negative effect while preserving the level of WT proteins [147]. The clustered regularly interspaced short palindromic repeat (CRISPR)/Cas9-mediated genome, a contemporary therapeutic paradigm, represents a revolutionary approach for irreversible suppression of mutant or aberrant proteins. This technique involves utilizing non-specific DNA-cleavage proteins, linked to a guide RNA, to effect precise DNA double-strand breaks in the identified mutant proteins [148,149,150]. The modulation of cellular signaling pathways governing the responses to catecholamines in cardiomyocytes, or the modulation of proteins interacting with *RyR2*, stands as a promising therapeutic approach, irrespective of the particular genetic mutation in question [22,151].

The evolving role of induced pluripotent stem cell (iPSC) technology in addressing clinical challenges associated with inherited channelopathies is noteworthy [152]. iPSC-derived cardiomyocytes (iPSC-CMs), a novel category of pluripotent cells derived through the reprogramming of human differentiated cells, possess the capacity to differentiate pathogenic mutations, enabling genotype and phenotype-guided risk stratification, as well as pharmacological management of channelopathies [153]. This avenue remains unexplored in CPVT studies to date. 

Despite the prevailing enthusiasm surrounding gene therapies, formidable challenges persist concerning the emergence of immune responses elicited by such interventions. An active area of investigation revolves around the triggering of deleterious innate immune responses, precipitating potentially lethal systemic inflammation, particularly observed in large animal models exposed to substantial doses of therapeutic cargo including viral vectors encoding foreign proteins [153]. Overcoming these challenges necessitates continued technical refinement of gene therapy platforms and accumulating nuanced experience in the intricate design and execution of clinical gene therapy trials in order to successfully bridge the gap from proof-of-concept to clinical translation.

## 9. Conclusions

CPVT is a rare but significant ion channelopathy, providing a crucial model for studying calcium-related arrhythmia mechanisms and advancing therapeutic developments. While the genetic basis and pathophysiology are well-understood, prompt diagnosis remains a challenge. Non-selective β-blocker therapy serves as the primary treatment but challenges such as poor compliance and inadequate dosage persist, underscoring the importance of patient education and constant re-evaluation. Flecainide, in combination with β-blockers, has shown effectiveness. Interventions like LCSD, RCSD or catheter ablation have shown promise in drug-refractory ventricular arrhythmias. While frequently selected for secondary prevention of cardiac arrest, ICDs are now advised only in specific cases due to persisting concerns about long-term device-related complications, particularly in pediatric population, underscoring the importance of further research and calls for clinician discretion when considering and prescribing such interventions. Despite significant progress, many unanswered questions persist, with a lack of robust evidence for interventions reflected in recent guidelines. Precision medicine emerges as the contemporary frontier in arrhythmology, leveraging personalized care with patient-specific induced pluripotent stem cells (iPSCs) and next-generation genome sequencing and editing. Advances in gene therapy offer promising prospects, particularly with efficient cardiomyocyte delivery methods. Multidisciplinary collaborations are imperative for integrating genomic information with clinical details, enabling personalized risk prediction and management for rare channelopathies. As we navigate this evolving landscape, choosing diagnostic and therapeutic tools judiciously on an individualized basis remains pivotal, aiming for both safety and an enhanced quality of life for these patients.

## Figures and Tables

**Table 1:** Main genetic and epidemiologic characteristics of different types of CPVT.

Type	Predominant Inheritance Pattern	Protein	Gene	Chromosome Locus	Prevalence of Probands (%)
CPVT 1	Autosomal dominant	Ryanodine receptor 2	RYR2	1q42-q43	60–70%
CPVT 2	Autosomal recessive	Calsequestrin 2	CASQ2	1p13.1	2–5%
CPVT 3	Autosomal recessive	Trans-2,3-enoyl-CoA-reductase-like	TECRL	7p22-p14	Rare (<1%)
CPVT 4	Autosomal dominant	Calmodulin 1	CALM1	14q31-q32	Rare (<1%)
CPVT 5	Autosomal recessive	Triadin	TRDN	6q22.31	Rare (<1%)
CPVT 6	Autosomal dominant	Calmodulin 3	CALM3	19q13.22	Rare (<1%)

**Table 2:** Expert consensus recommendations on diagnosis of CPVT.

CPVT: Expert Consensus Recommendations on Diagnosis
1. CPVT is diagnosed in the presence of a structurally normal heart, normal ECG and unexplained exercise or catecholamine induced bidirectional VT or polymorphic PVCs in patients < 40 years of age
2. CPVT is diagnosed in patients (index case or family member) who have a pathogenic mutation.
3. CPVT is diagnosed in family members of a CPVT index case with a normal heart who manifest exercise-induced PVCs or bidirectional/polymorphic VT.
4. CPVT can be diagnosed in the presence of a structurally normal heart and coronary arteries, normal ECG and unexplained exercise or catecholamine-induced bidirectional VT or polymorphic PVCs or VT in an individual > 40 years of age.

CPVT: Catecholaminergic polymorphic ventricular tachycardia; ECG: electrocardiogram; VT: ventricular tachycardia; PVC: premature ventricular complexes. From [54].

**Table 3:** Catecholaminergic polymorphic ventricular tachycardia diagnostic scorecard.

Clinical Criteria	Points
Symptoms	
Exercise/activity-associated ACA/SCA	2
Exercise/activity-associated syncope or generalized seizures	1
Exercise stress test or Holter monitoring during exertional activity (REQUIRES ≥ 1 exercise stress test/ambulatory Holter finding) *^†^	
Inducible bidirectional ventricular tachycardia at HR > 100 bpm	4
Inducible PVCs in bigeminy and bidirectional couplets at HR > 100 bpm	2
Inducible PVCs at HR > 100 bpm	1
Baseline HR QTc ^‡^	
QTc ≤ 420 ms	0.5
421 < QTc < 460 ms	0
QTc ≥ 460 ms	−0.5
CPVT genetic test	
Positive for ACMG-graded pathogenic variant	4
Positive for ACMG-graded likely pathogenic variant	2
Positive for a variant of uncertain significance	0
Negative CPVT genetic test (*RyR2*, *CASQ2*, *TRDN* and *CALM1-3*)	−1
Holter	
Ambulatory ventricular ectopy (>2% of total beats)	−1
Imaging (TTE or cardiac MRI/CT) ^§^	
Evidence of ischemic or structural heart disease	−2
Age	
≥50 y of age at time of sentinel event	−1
Family history *	
First-degree relative with definite CPVT	1.5
Suspicious autopsy-negative SCD (exertional, near drowning, etc.) in a first- or second-degree relative ≤ 45 years.	1
Unexplained autopsy-negative SCD in a first- or second-degree relative age ≤ 45 years.	0.5
**CPVT score (requires an exercise stress test/ambulatory Holter finding)**	
3.5–12 points: high pre-test probability of CPVT (definite/probable CPVT ≥ 90% likelihood)	
2–3 points: intermediate pre-test probability of CPVT (possible CPVT, ≈50% likelihood)	
0.5–1.5 points: low pre-test probability of CPVT (nondiagnostic)	
≤0 points: no evidence of CPVT	
No score: indeterminate	

* Only award points once for highest score within category. ^†^ In the absence of digitalis use/toxicity. ^‡^ Points should not be awarded for QTc values derived from ECGs obtained within one week of cardiac event (faint, cardiac arrest, etc.). ^§^ For calculating the CPVT score, imaging parameters defined as left ventricular ejection fraction 58 mm for females and >64 for males, RWMAs and presence of significant valvular heart disease. ACA: aborted cardiac arrest; ACMG: American College of Medical Genetics; CT: computed tomography; HR: heart rate; MRI: magnetic resonance imaging; PVC: premature ventricular contraction; QTc: corrected QT interval; RWMA: regional wall motion abnormalities; SCA: sudden cardiac arrest; SCD: sudden cardiac death; TTE: transthoracic echocardiogram. From [55].

## Data Availability

Not applicable.

## Abbreviations

ACA	Aborted cardiac arrest
ACMG	American college of medical genetics
AMP	Association for molecular pathology
Ca^2+^	Calcium
CASQ	Calsequestrin
CNV	Copy number variants
CPET	Cardiopulmonary exercise testing
CPVT	Catecholaminergic polymorphic ventricular tachycardia
CRISPR	Clustered regularly interspaced short palindromic repeat
CSD	Cardiac sympathetic denervation
CT	Computed tomography
EAPC	European association of preventive cardiology
EHRA	European heart rhythm association
EKG	Electrocardiogram
ERP	Early repolarization pattern
ESC	European society of cardiology
HCN	Hyperpolarization-activated cyclic nucleotide
ICD	Implantable cardioverter defibrillator
iPSC	Induced pluripotent stem cell
LBBB	Left bundle branch block
LCSD	Left cardiac sympathetic denervation
LV	Left ventricle
MRI	Magnetic resonance imaging
NSVT	Non-sustained ventricular tachycardia
PVC	Premature ventricular complexes
QTc	Corrected QT interval
RBBB	Right bundle branch block
RCSD	Right cardiac sympathetic denervation
RCT	Randomized controlled trials
RVOT	Right ventricular outflow tract
RWMA	Regional wall motion abnormalities
RyR	Ryanodine receptor
SCA	Sudden cardiac arrest
SCD	Sudden cardiac death
S-ICD	Subcutaneous implantable cardioverter defibrillator
SIDS	Sudden infant death syndrome
SNV	Single nucleotide variants
TdP	Torsades-de-Pointes
TECRL	Trans-2,3-Enoyl-CoA-Reductase-like
TRDN	Triadin
TTE	Transthoracic echocardiogram
VAT	Ventilatory anaerobic threshold
VF	Ventricular fibrillation
VT	Ventricular tachycardia
VUS	Variants of uncertain significance
WT	Wild-type
